# Resection of a pancreatic capillary lymphatic malformation through a partial pancreatectomy: a case report

**DOI:** 10.1186/s40792-019-0603-z

**Published:** 2019-03-28

**Authors:** Shigeto Ochiai, Koichi Tomita, Masashi Nakagawa, Itsuki Koganezawa, Kei Yokozuka, Takahiro Gunji, Kosuke Hikita, Yosuke Ozawa, Toshimichi Kobayashi, Toru Sano, Rina Tsutsui, Naokazu Chiba, Midori Wakiya, Hiroshi Hirano, Shigeyuki Kawachi

**Affiliations:** 1 Department of Digestive and Transplantation Surgery, Tokyo Medical University Hachioji Medical Center, 1163 Tatemachi, Hachiojishi, Tokyo 193-0998 Japan; 2 Department of Diagnostic Pathology, Tokyo Medical University Hachioji Medical Center, 1163 Tatemachi, Hachiojishi, Tokyo 193-0998 Japan

**Keywords:** Pancreatic vascular malformation, Capillary lymphatic malformation, Surgical resection

## Abstract

**Background:**

Pancreatic vascular malformation causes epigastric pain, pancreatitis, portal vein hypertension, bleeding, and rupture. It is a rare disease, with most pancreatic vascular malformations being arteriovenous malformations (AVMs) and the other types of malformations being rare. We report a case of capillary lymphatic malformation (CLM) in the pancreatic uncinate process.

**Case presentation:**

A 74-year-old woman, who presented with complaints of repeated upper abdominal pain, was admitted to our institution. Contrast-enhanced dynamic computed tomography (CT) scan revealed that the tumor in the pancreatic uncinate process had a poor contrast effect in the arterial phase and a small contrast effect in the equilibrium phase, which are suggestive of a benign disease-like vascular malformation. However, we suspected that it could possibly be a malignant tumor because the tumor size tended to increase over time; thus, we decided to perform a surgery. We resected the tumor through a partial resection of the pancreas. Macroscopically, the cut surface of the tumor had a spongioid appearance. Histopathological examination findings showed a mixed shape of small capillaries and lymphatic ducts. The patient was diagnosed with CLM according to the International Society for the Study of Vascular Anomalies (ISSVA) classification, based on the histological appearance and immunostaining findings. The postoperative course of the patient was uneventful.

**Conclusions:**

We reported a case of pancreatic vascular malformation, specifically a CLM, which was completely resected through a partial pancreatectomy.

## Background

Pancreatic vascular malformation was first reported in 1968 by Halpern et al. [1]. Though it is a benign disease, it is clinically important because it can cause epigastric pain, pancreatitis, portal vein hypertension, bleeding, and rupture [2]. However, pancreatic vascular malformation is a rare disease [3] despite the advancement of various imaging modalities [4]. Moreover, most of the pancreatic vascular malformations are arteriovenous malformations (AVMs), with the other types of malformations being rare. The current report describes a patient with capillary lymphatic malformation (CLM) in the pancreatic uncinate process, which was completely resected through a partial pancreatectomy.

### Case presentation

A 74-year-old woman, who presented with complaints of repeated upper abdominal pain for 3 days, was admitted to our hospital. She had no relevant past medical history. Abdominal ultrasonography and computed tomography (CT) scan at another hospital revealed a tumor in the pancreatic uncinate process; thus, she was referred to our hospital for a comprehensive examination.

The results of the laboratory tests were found to be almost normal (the italicized text indicates the test results with abnormal values): *white blood cell 9130/μl*, total bilirubin 1.0 mg/dl, aspartate aminotransferase 16 U/l, alanine aminotransferase 9 U/l, hemoglobin A1c 6.0%, amylase 76 U/l, C-reactive protein < 0.02 mg/dl, Ca 10.3 mg/dl, soluble interleukin-2 receptor 271.0 U/ml, IgG4 31.5 mg/dl, and antinuclear antibody 160 index. The levels of tumor markers were also normal: carcinoembryonic antigen 3.0 ng/mL, carbohydrate antigen 19-9 11.0 U/ml, DUPAN-2 < 25 U/ml, span-1 8.9 U/ml, and elastase-1 85 ng/dl.

**Fig. 1:**
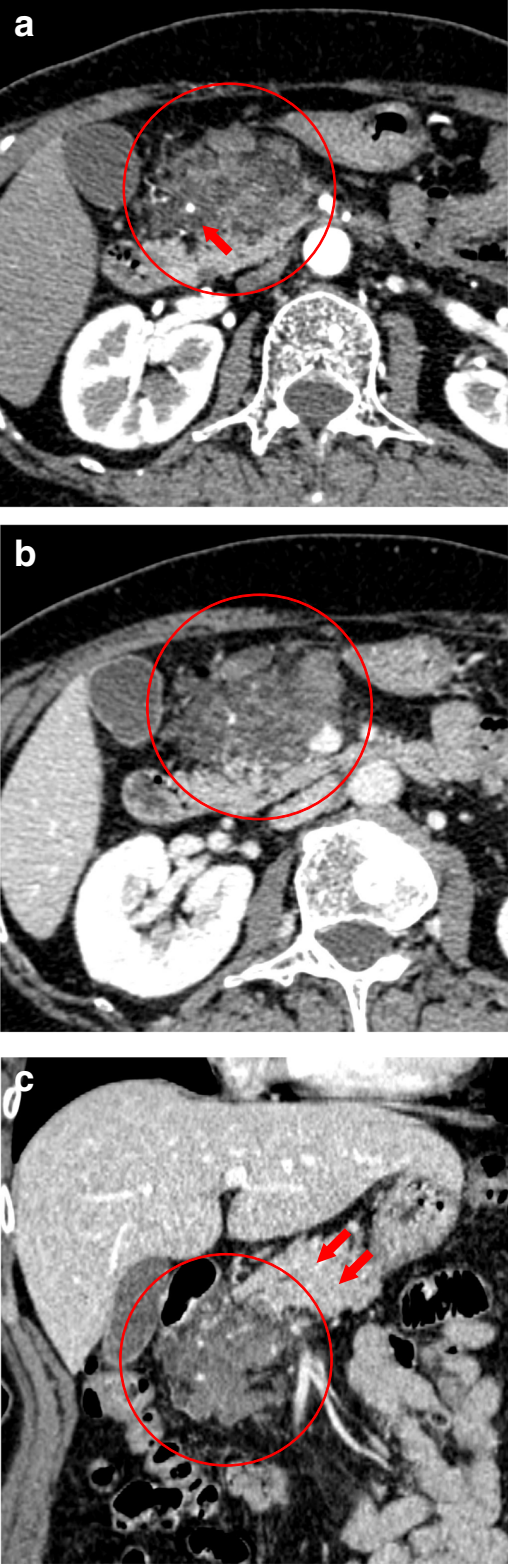
The contrast-enhanced dynamic CT scan showed a 60-mm-diameter tumor in the pancreatic uncinate process (red circle in each image). **a** Arterial phase (red arrow: gastroduodenal artery). **b**, **c** Portal vein phase (red arrow: normal pancreas)

An abdominal contrast-enhanced dynamic CT scan showed a 60-mm-diameter tumor in the pancreatic uncinate process, accompanied by multiple cysts (Fig. 1), and the tumor size tended to increase over time. The gastroduodenal artery was noted to be passing through within the tumor. The pancreatic duct was not enlarged and separated from the tumor. The tumor had a poor contrast effect in the arterial phase and a small contrast effect in the equilibrium phase.

**Fig. 2:**
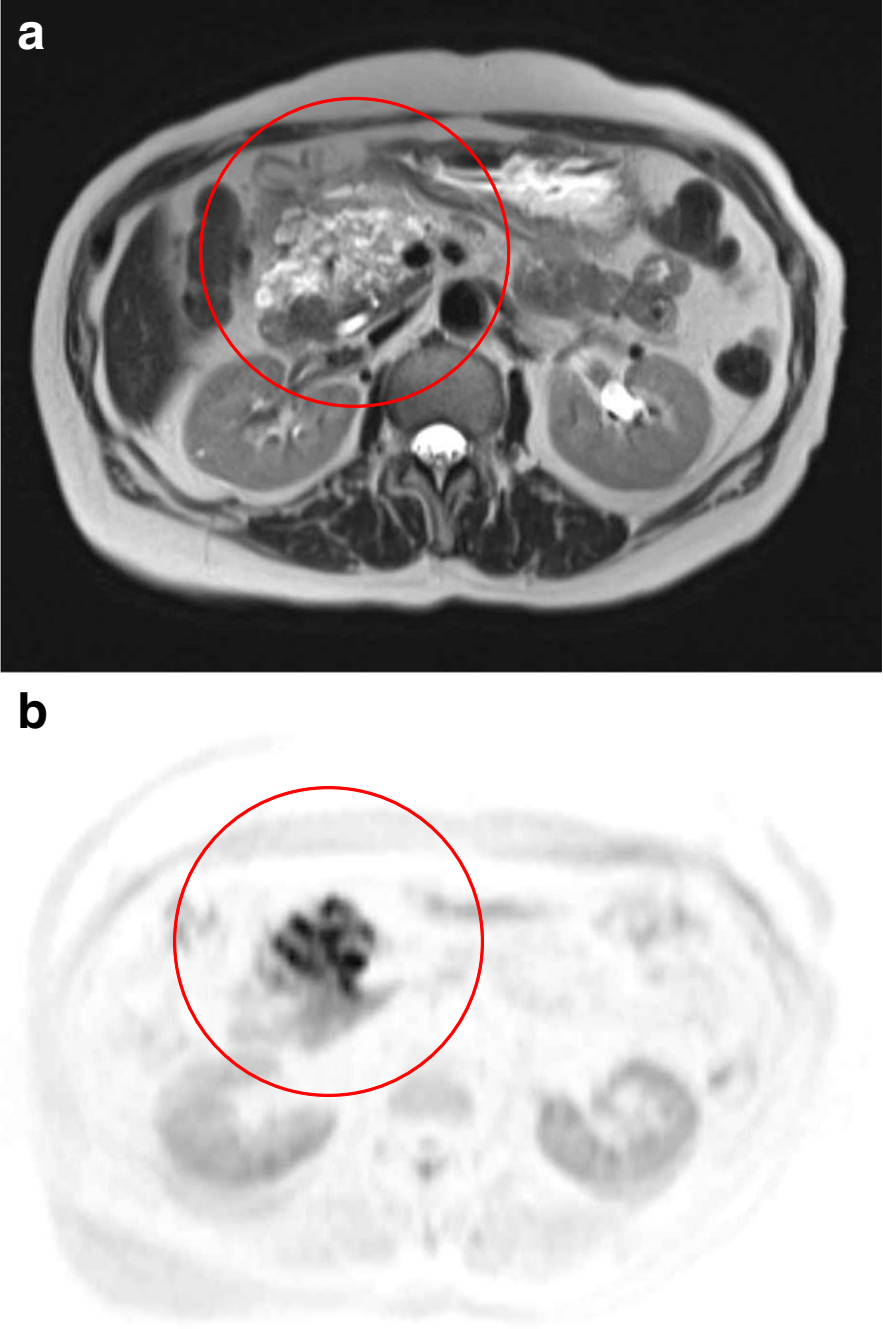
MRI showed a lobulated tumor with mixed high and low signals on T2-weighted imaging (**a**). Diffusion-weighted images revealed a high signal lobulated tumor (**b**). (red circle in each image)

The gadolinium-ethoxybenzyl-diethylenetriamine pentaacetic acid-enhanced magnetic resonance imaging (MRI) showed a lobulated tumor with mixed high and low signals on T2-weighted imaging (Fig. 2a). Out-of-phase T1-weighted imaging showed a low-intensity area, which was a fat component. This finding suggested that the tumor was unlikely to be a malignant tumor. However, diffusion-weighted images revealed a high signal lobulated tumor and suggested potential for malignancy (Fig. 2b). Magnetic resonance cholangiopancreatography revealed a soft tissue tumor close to the pancreatic uncinate process, and it was not continuous with the main pancreatic duct.

**Fig. 3:**
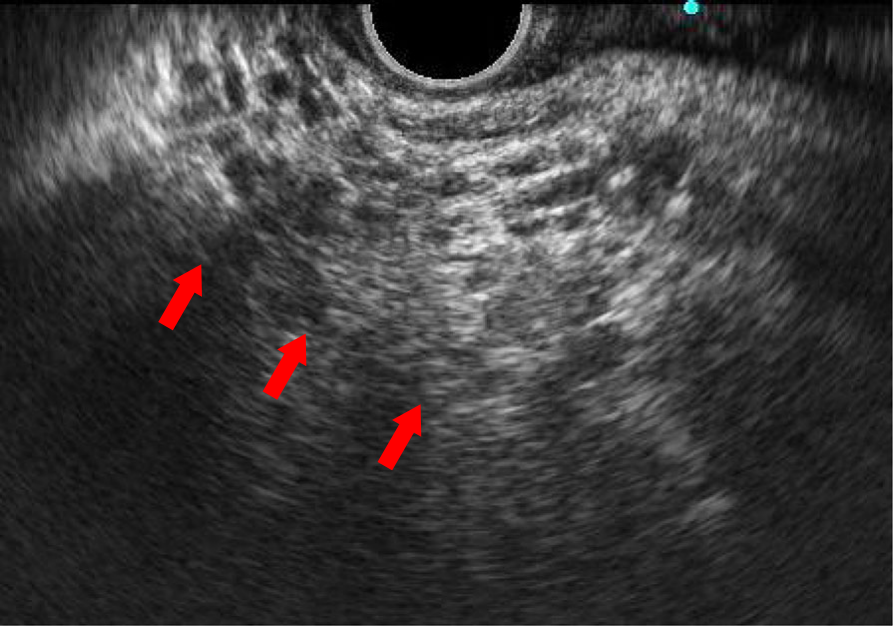
EUS showed a collective cystic lesion on the pancreatic uncinate process (red arrow)

An upper gastrointestinal examination revealed that the gastric angle was pushed to the dorsal side of the stomach by the tumor. Endoscopic ultrasonography (EUS) showed a collective cystic lesion on the ventral side of the pancreatic uncinate process (Fig. 3). The main pancreatic duct was negative for intraductal papillary mucinous neoplasm. In addition, given that the tumor was accompanied by a cystic lesion, a fine-needle aspiration was not performed.

In the positron emission tomography CT scan, there was no significant fluoro-deoxy-glucose accumulation in the soft tissues of the pancreatic uncinate process.

The tumor in the pancreatic uncinate process was thought to be the possible cause of the upper abdominal pain. Based on the abovementioned findings, we considered that the tumor was a benign vascular malformation, but because the tumor size tended to increase over time, there was a possibility of it being a malignant tumor; thus, we planned on performing a pancreatoduodenectomy (PD).

After a detailed examination, we performed an elective open laparotomy. During the operation, intraoperative findings revealed that the tumor appeared to be benign and was separate from the pancreatic duct or bile duct. We therefore performed a partial pancreatectomy instead of PD. Since the right gastroduodenal artery and small vein penetrated the tumor, they were ligated and detached. The tumor was excised with a small part of the pancreatic uncinate process, and the mesentery of the transverse colon was also removed. The operation time was 200 min with 75 ml blood loss. The patient’s postoperative course was uneventful, and she was discharged on postoperative day 12.

**Fig. 4:**
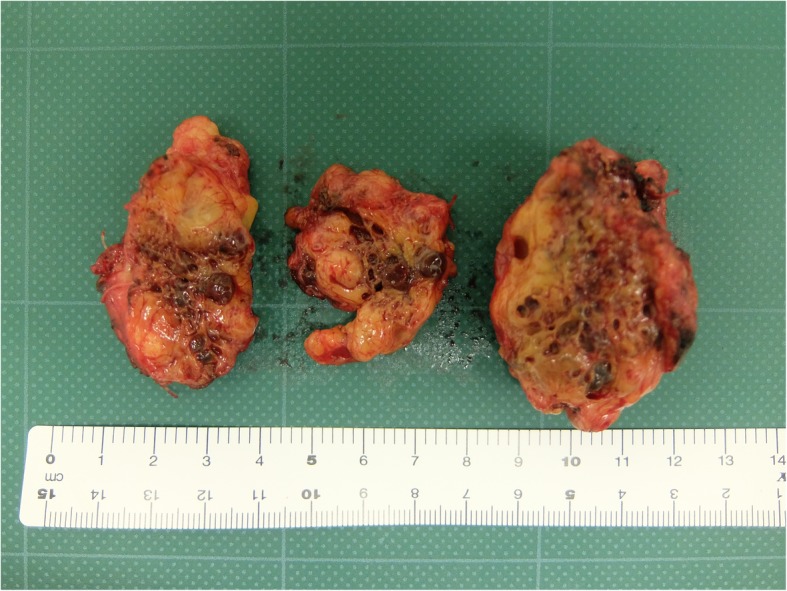
Macroscopically, the tumor showed spongioid appearance of the cut surface

**Fig. 5:**
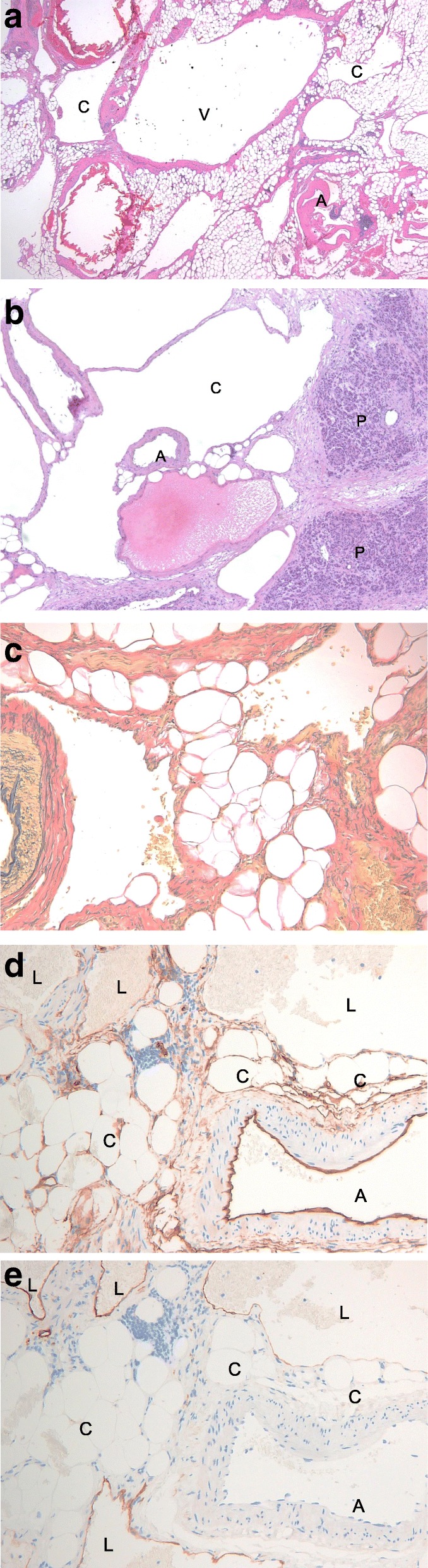
The histopathological findings showed complex multiple small vessels (**a**) (A, artery; V, vein; C, capillary). The pancreatic tissue was recognized within the tumor (**b**) (A, artery; C, capillary; P pancreas). Elastica van Gieson staining was negative for most small vessels (**c**). CD34 was positive for capillaries including a small artery (**d**) (A, artery; C, capillary L; lymphatic duct). D2-40 staining was positive for only the lymphatic ducts (**e**) (A, artery; C, capillary; L, lymphatic duct)

Macroscopically, the tumor was a 58 × 46 × 30-mm specimen with a spongioid appearance of the cut surface (Fig. 4). Histologically, hematoxylin and eosin staining showed a mixed shape of small veins, small arteries, and capillaries (Fig. 5). The pancreatic tissue was recognized within the tumor, suggesting that the tumor originated from the pancreas. There was no lesion with suspected malignancy. We performed immunostaining for CD31, CD34, Factor VIII, and D2-40, which revealed the following in general: CD31 was positive for vascular endothelium and histiocyte; CD34 and Factor VIII were positive for vascular endothelium; and D2-40 was positive for lymphatic endothelium. In the Elastica van Gieson staining, most vessels of the tumor had no muscular layer. These vessels were capillary blood vessels (CD31, CD34, and Factor VIII were positive, and D2-40 was negative) and lymphatic vessels (CD31 and D2-40 were positive; CD34 and Factor VIII were negative). Based on both histological appearance and immunostaining findings, we diagnosed the tumor as a capillary lymphatic malformation (CLM) according to the International Society for the Study of Vascular Anomalies (ISSVA) classification.

## Discussion

The incidence of pancreatic vascular malformation is extremely low. In previous reports, the common sites of AVM in the digestive organs are as follows: cecum and ascending colon (78%), jejunum (10.5%), ileum (5.5%), duodenum (2.3%), stomach (1.4%), and pancreas (0.9%) [3]. Most patients with pancreatic vascular malformation are diagnosed because they experience abdominal pain, gastrointestinal bleeding, and pancreatitis; however, 22% of the patients are asymptomatic [5] and are only diagnosed by chance. According to another report, pancreatic vascular malformations are localized in the head (57%) and the body and tail (31%) of the pancreas [5]. The current case was diagnosed, due to epigastric pain, as having a pancreatic vascular malformation localized in the head of the pancreas.

According to the ISSVA classification, vascular malformations can be classified as simple or combined malformation [6]. The simple vascular malformation can be classified as capillary malformation (CM), lymphatic malformation (LM), venous malformation (VM), and arteriovenous malformation (AVM). The combined vascular malformation is the combination of simple vascular malformations. The malformation in this report was considered as a CLM, which is a complex-combined malformation of both capillary and lymphatic malformations. Most of the pancreatic vascular malformation is AVM, with the other types of malformation being rare.

The typical contrast-enhanced dynamic CT images of pancreatic AVM show a strong enhancement of the small hypervascular spots in the tumor and early contrast filling of the small veins or portal vein. However, in the current case, the contrast-enhanced dynamic CT scan prior to the surgery revealed that most of the tumor was not adequately enhanced, and there were a few arteries and veins within the tumor. This was confirmed by the histopathological examination of the tumor, which showed a number of vessels or duct-like structures. Immunostaining showed that the tumor was positive for CD 31 and D2-40 and was negative for CD34 and Factor VIII. All of these are vascular markers, and D2-40 is specific for the lymphatic duct. This result showed that the vessels or duct-like structures were capillary vessels and lymphatic ducts. In our literature search, we could not find any case report of CLM of the pancreas in the past. The current case may be the first report of pancreatic CLM.

In terms of the management for pancreatic vascular malformations, the best treatment option is surgical resection of the tumor, especially in case of being symptomatic or having a possibility of malignancy. PD is mostly performed for pancreatic vascular malformations [7]. Transcatheter arterial embolization is also considered as a treatment for this condition [8]; however, it can be performed only for arterial dominant tumor (not in this case), and its success rate varies between reports [9]. If there is an indication, surgical resection is considered to be the best approach for complete curative management of pancreatic vascular malformation, though it carries the risk of massive intraoperative bleeding. In our case, we were able to excise the tumor through a partial resection of the pancreas with little bleeding.

## Conclusion

We reported a case of pancreatic vascular malformation, specifically a CLM, which was completely resected through a partial pancreatectomy.

## Abbreviations

AVMs: Arteriovenous malformations
CLM: Capillary lymphatic malformation
CT: Computed tomography
EUS: Endoscopic ultrasonography
ISSVA: The International Society for the Study of Vascular Anomalies
MRI: Magnetic resonance imaging
PD: Pancreatoduodenectomy
